# The Role of PPARβ/δ in Melanoma Metastasis

**DOI:** 10.3390/ijms19102860

**Published:** 2018-09-20

**Authors:** Jonathan Chee Woei Lim, Yuet Ping Kwan, Michelle Siying Tan, Melissa Hui Yen Teo, Shunsuke Chiba, Walter Wahli, Xiaomeng Wang

**Affiliations:** 1 Lee Kong Chian School of Medicine, Nanyang Technological University Singapore, 59 Nanyang Drive, Singapore 636921, Singapore; cheewoei@upm.edu.my (J.C.W.L.); kwanyuetping@ntu.edu.sg (Y.P.K.); michelle.siying@ntu.edu.sg (M.S.T.); teohy@imcb.a-star.edu.sg (M.H.Y.T.); 2 Department of Medicine, Faculty of Medicine and Health Sciences, Universiti Putra Malaysia, Serdang 43400, Selangor, Malaysia; 3 Division of Chemistry and Biological Chemistry, School of Physical and Mathematical Sciences, Nanyang Technological University, 21 Nanyang Link, Singapore 637371, Singapore; Shunsuke@ntu.edu.sg; 4 Center for Integrative Genomics, University of Lausanne, Le Génopode, 1015 Lausanne, Switzerland; 5 Institute of Molecular and Cell Biology, Agency for Science, Technology and Research (A*STAR), Proteos, 61 Biopolis Dr, Singapore 138673, Singapore; 6 Singapore Eye Research Institute, The Academia, 20 College Road, Discovery Tower Level 6, Singapore 169856, Singapore; 7 Institute of Ophthalmology, University College London, 11-43 Bath Street, London EC1V 9EL, UK

**Keywords:** melanoma, peroxisome proliferator–activated receptor β/δ, migration, EMT, invasion, metastasis

## Abstract

Background: Peroxisome proliferator–activated receptor (PPAR) β/δ, a ligand-activated transcription factor, is involved in diverse biological processes including cell proliferation, cell differentiation, inflammation and energy homeostasis. Besides its well-established roles in metabolic disorders, PPARβ/δ has been linked to carcinogenesis and was reported to inhibit melanoma cell proliferation, anchorage-dependent clonogenicity and ectopic xenograft tumorigenicity. However, PPARβ/δ’s role in tumour progression and metastasis remains controversial. Methods: In the present studies, the consequence of PPARβ/δ inhibition either by global genetic deletion or by a specific PPARβ/δ antagonist, 10h, on malignant transformation of melanoma cells and melanoma metastasis was examined using both in vitro and in vivo models. Results: Our study showed that 10h promotes epithelial-mesenchymal transition (EMT), migration, adhesion, invasion and trans-endothelial migration of mouse melanoma B16/F10 cells. We further demonstrated an increased tumour cell extravasation in the lungs of wild-type mice subjected to 10h treatment and in *Pparβ/δ^−/−^* mice in an experimental mouse model of blood-borne pulmonary metastasis by tail vein injection. This observation was further supported by an increased tumour burden in the lungs of *Pparβ/δ^−/−^* mice as demonstrated in the same animal model. Conclusion: These results indicated a protective role of PPARβ/δ in melanoma progression and metastasis.

## 1. Introduction

Melanoma is the deadliest and the most aggressive form of skin cancer that originates from the pigment making melanocytes in epidermis. In contrast to the declining incidence for many types of cancers, the number of annually diagnosed melanoma cases has doubled in the past decade [1]. Furthermore, melanoma is more likely to spread than any other types of skin cancers and the lung is the most common site of distant metastases [2]. Although surgical treatment of early-stage melanoma shows a 90% cure rate, therapeutic options for advanced melanoma are very limited. To make things worse, melanoma cells are particularly adept at rewiring themselves and will inevitably evolve resistance to treatments [3]. The median survival for patients with metastatic malignant melanoma ranges from 6–15 months [4] and the 5-year survival rate is only around 5% [5]. To date, effective therapies for metastatic melanoma remain a significant challenge.

Peroxisome proliferator–activated receptors (PPARs) are ligand-activated transcription factors that belong to the nuclear receptor superfamily. They regulate many biological processes, including cell proliferation, migration and differentiation, immune response, and energy homeostasis [6]. Upon ligand binding, PPARs undergo conformational change which allows the release of corepressors, recruitment of coactivators and subsequent activation of downstream target genes. There are three distinct members of the PPAR family: PPARα, PPARβ/δ, and PPARγ [7]. The biological roles of PPARα and PPARγ have been extensively studied, whereas, PPARβ/δ’s function is less clear or even controversial. Several studies linked PPARβ/δ to tumour growth and progression with both promoting and inhibitory effects reported [8,9,10,11]. PPARβ/δ was previously shown to be expressed in melanocytes [12], suggesting a potential physiological role in melanocyte activity and function. Although the activation of PPARβ/δ has no impact on melanocyte proliferation [12], it significantly inhibits the proliferation of melanoma cells, prevents anchorage-dependent clonogenicity and attenuates ectopic xenograft tumorigenicity [13]. On the contrary, shRNA-mediated knockdown of PPARβ/δ in a highly malignant mouse melanoma cell line B16-F10 demonstrated reduced lung metastasis and tumour burden [9]. In this study, we showed that PPAβ/δ inhibition by either the PPAβ/δ antagonist 10h, or gene deletion promotes the transformation of melanoma cells towards a more malignant phenotype, leading to increased melanoma cell extravasation and tumour burden in the lungs.

## 2. Results

### 2.1. 10h Induces Phenotypic Changes of B16/F10 Mouse Melanoma Cells

**Figure 1:**
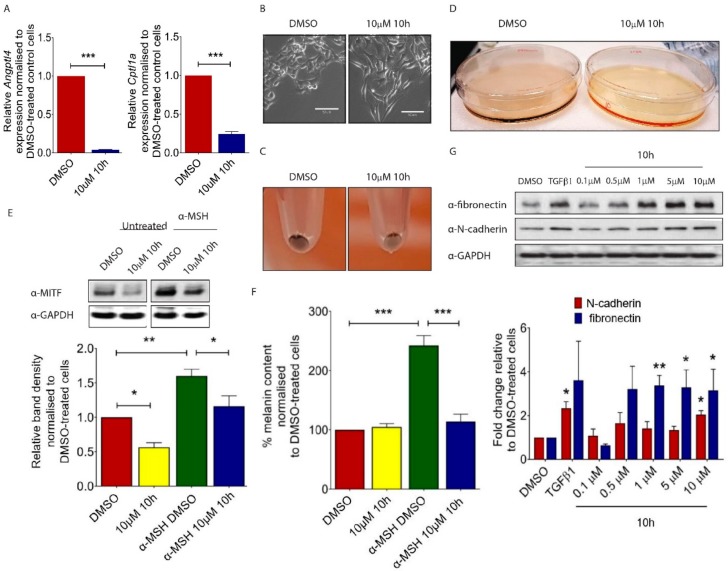
Effect of 10h on B16/F10 mouse melanoma cells. (**A**) *Angptl4* and *Cpt1* gene expression measured using real-time quantitative PCR analysis. (**B**) Morphology of B16/F10 cells after treatment with 10 µM of 10h in 5% serum supplemented DMEM compared to 0.05% DMSO-treated control cells. Scale bar: 50 µm. Representative picture of trypsinized B16/F10 cell pellets (**C**) and conditioned medium (**D**) after 72 h treatment with 10 µM of 10h. (**E**) Representative images and quantitative analysis of western blot for MITF in α-MSH and/or 10h-treated B16/F10 melanoma cells. (**F**) Percentage of melanin content in α-MSH and/or 10h-treated B16/F10 melanoma cells. (**G**) Representative images and quantitative analysis of western blot for fibronectin, N-cadherin, and GAPDH in 10h-treated B16/F10 cells. Data are presented as mean ± s.e.m of three independent experiments. Statistical analysis was performed using one-way ANOVA followed by Turkey’s post hoc analysis or two-tailed, unpaired student’s *t*-test; * *p* < 0.05, ** *p* < 0.01, *** *p* < 0.001.

Although melanoma cells do not show classic epithelial or mesenchymal characteristics, they undergo a coordinated phenotypic switch which is necessary for tumour cells to disperse from the primary site [14]. To evaluate the role of PPARβ/δ in melanoma cell function, we used a novel PPARβ/δ antagonist 10h [15,16]. 10h has been shown to efficiently antagonized agonist-mediated transcriptional activation of PPARβ/δ. On the contrary, it had no significant effect on ligand activated PPARα or PPARγ [15]. To test the efficacy of 10h-mediated PPARβ/δ inhibition, we first evaluated the expression of known PPARβ/δ target genes in 10h-treated B16/F10 cells and demonstrated a significant reduction in Angiopoietin-like 4 (*Angptl4*) and *Cpt1* expression (Figure 1A). ANGPTL4 was previously demonstrated to prevent tumour metastasis by inhibiting tumour cell motility and invasiveness [17]. Consistent with this observation, 10h-treated B16/F10 cells underwent a drastic change in morphology and were converted from a typical “cuboidal” shape into an elongated mesenchymal like structure (Figure 1B). This phenotypic change was associated with an apparent depigmentation in both the 10 h-treated B16/F10 cells (Figure 1C) and conditioned medium of these cells (Figure 1D), which are characteristic features of transformed invasive melanoma cells [18]. Microphthalmia-associated transcription factor (Mitf) drives the expression of a number of genes involved in melanocyte pigmentation [19]. The expression of this factor is stimulated by the α-melanocyte-stimulating hormone (α-MSH), an endogenous peptide hormone that plays a critical role in melanogenesis. Our study showed that 10h significantly attenuated both basal and α-MSH-induced Mitf expression in B16/F10 cells (Figure 1E). Consistently, there was a significant reduction in the α-MSH-induced melanin secretion after 10h treatment (Figure 1F). Transforming growth factor (TGF) β1 is a potent stimulator of epithelial to mesenchymal transition (EMT) during tumour invasion and metastasis [20]. Similarly to TGFβ1, 10h significantly induced the expression of the specific mesenchymal markers Fibronectin and N-cadherin in B16/F10 cells (Figure 1G). Together, our study showed that 10h induces the switch of melanoma cells towards a more transformed phenotype.

### 2.2. 10h Promotes Melanoma Cell Migration and Invasion

**Figure 2:**
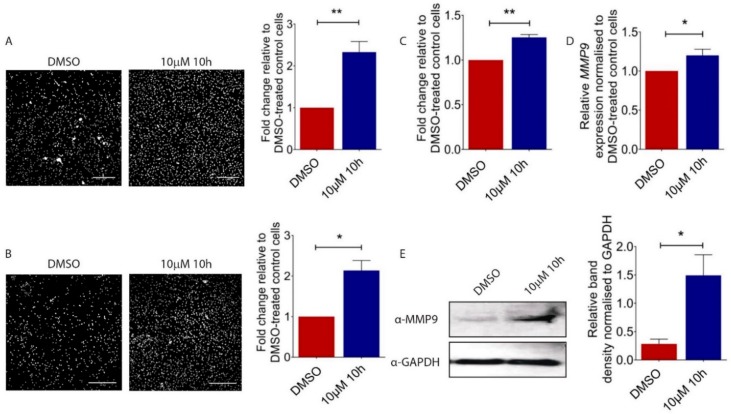
Effect of 10h on B16/F10 cell migration and invasion. (**A**) Representative images and quantitative analysis of migrated B16/F10 cells after 10h and DMSO treatments. (**B**) Representative images and quantitative analysis of invading B16/F10 cells after respective treatments. Scale bar: 200 µm. (**C**) Quantitative analysis of B16/F10 cells attached to fibronectin coated plate normalised to 0.05% DMSO-treated controls. (**D**) MMP9 gene expression in 10 µM 10h or DMSO-treated B16/F10 cells as determined by real-time quantitative PCR analysis. (**E**) Representative Western blot and densitometry analysis of MMP9 in 10 µM 10h or 0.05% DMSO-treated control B16/F10 cells. All images are representative. Data are presented as the mean ± s.e.m of three independent experiments. Statistical analysis was performed using two-tailed, unpaired student’s *t*-test; * *p* < 0.05, ** *p* < 0.01.

To understand the functional consequences of the 10h-induced morphological transformation of melanoma cells, we carried out the Transwell migration assay and demonstrated an increased motility of 10 µM of 10h-treated B16/F10 cells as compared to vehicle-treated control cells (Figure 2A). Next, to mimic the invasion process, 10h-treated B16/F10 cells were seeded on top of a Matrigel coated Transwell membrane. Consistent with the increased motility, 10h significantly increased the invasiveness of B16/F10 cells (Figure 2B). During invasion, epithelial-derived tumour cells move from the lamina-enriched basal membrane to the collagen and fibronectin-enrich connective tissue region [21,22]. The ability of tumour cells to adapt to this abrupt change in microenvironment contributes to their metastatic and invasive behaviour. Consistently, our study showed a promoting effect of 10h on the capability of B16/F10 cells to adhere to fibronectin-coated cell culture plates (Figure 2C). A critical prerequisite for metastatic tumour cells to invade the surrounding tissue is their capacity to degrade extracellular matrix (ECM) by the action of matrix metalloproteinases (MMPs) [23,24,25]. Among all MMPs, MMP9 is particularly important for melanoma progression [26], and increased expression and activity of these MMPs were observed in invasive melanoma cell lines [27,28]. Our study showed that both transcript (Figure 2D) and protein (Figure 2E) levels of MMP9 were induced in 10h-treated B16/F10 cells. Together, our data showed a promoting effect of 10h on B16/F10 melanoma cell motility, invasion, and MMP9 expression, all critical characteristics for melanoma progression and metastasis. 

### 2.3. 10h Increases B16/F10 Melanoma Cell Adhesion to the Endothelium and Trans-Endothelial Migration

**Figure 3:**
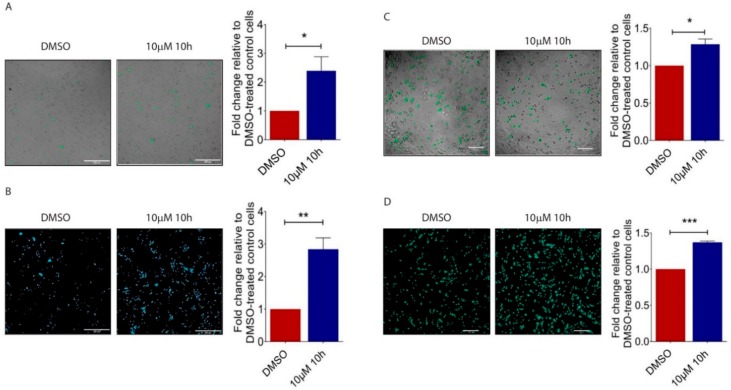
Impact of 10h on B16/F10 cell adhesion to endothelial cells and transendothelial migration. (**A**) Representative images and quantitative analysis of the number of CMFDA-positive B16/F10 cells adhered on the HPMEC monolayer. Scale bar: 200 µm. (**B**) Representative images and quantitative analysis of the number of CMFDA-positive cells that migrated across the HPMEC monolayer. Scale bar: 200 µm. (**C**) Representative images and quantitative analysis of the number of CMFDA-positive B16/F10 cells adhered on the 0.05% DMSO or 10 µM 10h-treated HPMEC monolayer. (**D**) Representative images and quantitative analysis of the number of CMFDA-positive cells that migrated across 0.05% DMSO- or 10 µM 10h-treated HPMEC monolayers. Scale bar: 200 µm. All images are representative. Data are presented as mean ± s.e.m of three independent experiments. Statistical analyses were performed using two tailed, unpaired student’s *t*-test. * *p* < 0.05; ** *p* < 0.01; *** *p* < 0.001.

To reach distant sites, cancer cells must be able to adhere to endothelial cells (ECs) and migrate across the endothelium [29,30]. To understand PPARβ/δ’s role in this process, we applied 10h treated B16/F10 cells to pulmonary microvascular endothelial cells (HPMECs) and observed an increased number of 10h-treated B16/F10 cells adhered to a HPMEC monolayer (Figure 3A). In vitro trans-endothelial migration assay is commonly used to mimic the process of tumour cells to cross the endothelium, a critical step in metastatic spread. Similarly, 10h significantly increased the number of 10h-treated B16/F10 cells that migrated across the HPMEC monolayer as compared to those treated with DMSO (Figure 3B). We then investigated the effects of PPARβ/δ inhibition in HPMECs on melanoma cell behaviour. B16/F10 cells were layered on top of the HPMECs pre-treated with 10h. Similarly to the above observation, there was an increased number of melanoma cells that adhered to (Figure 3C) and migrated across the 10h-treated HPMECs (Figure 3D). Together, these data demonstrated that PPARβ/δ inactivation by 10h either in tumour cells or in ECs facilitates tumour cell dissemination.

### 2.4. PPARβ/δ Inhibition Promotes Lung Metastasis of Melanoma Cells In Vivo

**Figure 4:**
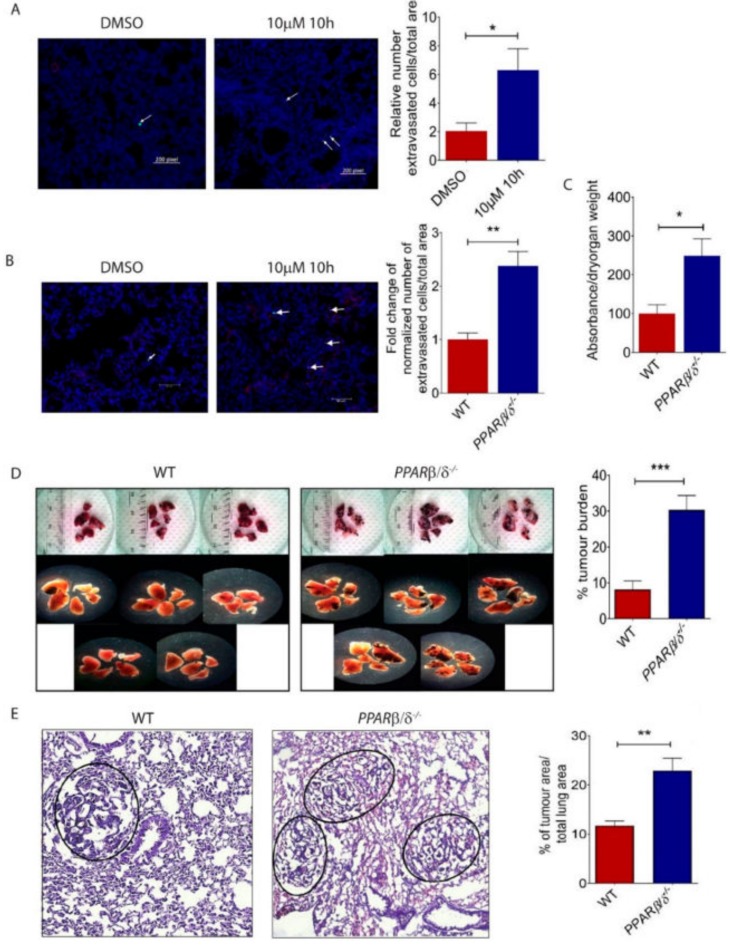
PPARβ/δ inhibition promotes pulmonary lung metastasis of B16/F10 melanoma cells. (**A**) Representative images and quantitative analysis of extravasated B16/F10 cells in C57BL/6 mice subjected to intravenous delivery of 10h- or vehicle-treated B16/F10 cells (*n* = 3). (**B**) Representative images and quantification of extravasated B16/F10 cells in the lungs of wild type (*n* = 6) or *Pparβ/δ^−/−^* mice (*n* = 7). Scale bar: 60 µm. Arrows indicate extravasated tumour cells. (**C**) Quantification of Evans blue dye extravasation in the lungs of wild type (*n* = 4) and *Pparβ/δ^−/−^* (*n* = 4) 30 min after Evans blue injection. (**D**) Representative images and quantification of the metastatic burden in the lungs of wild type (*n* = 8) and *Pparβ/δ^−/−^* mice (*n* = 8) subjected to intravenous delivery of B16/F10 cells. (**E**) Representative images and quantification of Hematoxylin & Eosin staining showing metastatic tumour nodules in the lungs of wild type (*n* = 5) and *Pparβ/δ^−/−^* mice (*n* = 5). All images are representative. Data are presented as the mean ± s.e.m. Significance was determined by two tailed, unpaired, Student’s *t*-test; * *p* < 0.05; ** *p* < 0.01; *** *p* < 0.001.

Having established an important role of PPARβ/δ in attenuating the morphological and functional transition of B16/F10 cells towards a more aggressive phenotype, we next investigated its involvement in lung metastasis in vivo. We observed an increased pulmonary extravasation of 10h-treated B16/F10 cells in an experimental C57BL/6 mouse model of blood-borne pulmonary metastasis, as compared to mice injected with DMSO-treated control cells (Figure 4A). Similarly, there was an increased extravasation of B16/F10 cells in *Pparβ/δ^−/−^* mice as compared to that in wild-type controls (Figure 4B). Furthermore, the lungs of *Pparβ/δ^−/−^* mice were leakier than WT counterparts as demonstrated by the Mile’s assay (Figure 4C). We further demonstrated an increased tumour burden in the lungs of *Pparβ/δ^−/−^* mice (Figure 4D,E). Together, these data showed that PPARβ/δ inhibition in tumour cells or *Pparβ/δ* deletion in the host promoted melanoma metastasis.

## 3. Discussion

Melanoma is the deadliest form of skin cancer. It is well known for its aggressive, metastatic and invasive properties [31], and its capability of rewiring itself to develop resistance to treatments [3]. Currently, treatments for metastatic melanoma are very limited and great efforts have been made towards developing novel targeted therapies.

PPARs are ligand-activated transcription factors that are members of the nuclear receptor superfamily. There are three related isotypes of PPARs, PPARα, PPARβ/δ, PPARγ, which share high levels of sequence homology but have distinct ligand specificity and tissue distribution patterns [32,33]. PPARs play numerous physiological functions and their dys-regulation is implicated in various pathological conditions, including cancers [34]. The roles of PPARβ/δ in cancer development and progression are very complicated with both promoting and protective roles reported [35,36]. For example, PPARβ/δ knockdown by shRNA in B16/F10 cells has been shown to significantly attenuate lung metastasis [9] and the ligand-mediated PPARβ/δ activation by GW501516 is able to increase the migration and invasion of the highly metastatic human melanoma cell line A375SM [31]. However, GW501516 was also reported to reduce metastasis and migration of pancreatic cancer cells, whereas short hairpin RNA-mediated inhibition of PPARβ/δ has been shown to promote the invasiveness of pancreatic cancer cells [37]. Furthermore, GW501516 is able to inhibit the expression of MMP-9, an important matrix remodelling proteinase that is involved in degrading pericellular and stromal compartments to facilitate metastasis [38]. Our study suggests a protective instead of promoting role of PPARβ/δ in melanoma progression and metastasis. Inhibition of PPARβ/δ signalling by 10h leads to phenotypic transformation of melanoma cell B16/F10 to elongated mesenchymal like shaped mimicking the initiation of EMT, an important process for tumour cells to achieve further differentiation and progress to an advanced stage [39]. This observation is supported by a reduced expression of melanocyte lineage-specific transcription factor, Mitf, a key regulator of melanin synthesis [40,41]. In line with this observation, 10h-treated B16/F10 cells and conditioned medium from these cells showed a significant reduction in pigmentation. This is associated with a concomitant increase of mesenchymal markers, fibronectin and N-cadherin. Besides its role in phenotypic expression of the melanocytic lineage, Mitf is reported to act as a suppressor of melanoma invasion and metastasis [42,43,44,45]. Consistent with the reduced Mitf expression, 10h promotes B16/F10 cell migration, expression of MMP9, invasion into the Matrigel, and adhesion to fibronectin. To metastasize to distant organs, cancerous cells need to extravasate from the vascular system into the surrounding tissue [46]. Our data demonstrate that PPARβ/δ inactivation in both B16/F10 cells and PMVECs promotes melanoma cell adhesion to and transmigration through the endothelium. We further demonstrated increased extravasation of 10h-treated B16/F10 cells in C57/BL6 mice in an experimental mouse model of blood-borne pulmonary metastasis. Similar observation was made in *Pparβ/δ*^−/−^ injected with B16/F10 cells. Finally, *Pparβ/δ* deletion in host tissue caused increased pulmonary vessel leakage and tumour burden in the lungs. In summary, our data support a critical role of PPARβ/δ in attenuating melanoma progression and metastasis in various in vitro and in vivo models.

However, each experimental model has its own limitations and could not faithfully recapitulate all characteristic features of tumour development and progression, particularly the role played by the different cell types, such as the predominant cancer-associated fibroblasts (CAFs), tumour associated macrophages (TAMs), tumour-infiltrating lymphocytes (TILs) and pericytes in tumour progression [47]. Furthermore, levels of PPARβ/δ inhibition or activation by small molecule drugs, combination of ligand target and off-target effects, subtle differences in experimental design, may also contribute to the controversial observations on PPARβ/δ’s role in tumourigenesis. Much more research will be necessary to clarify the multifaceted and intriguing pro- and anti-tumorigenesis roles of PPARβ/δ in different cancers.

## 4. Material and Methodology

### 4.1. Animals

*Pparβ/δ*-null mice (mixed genetic background of Sv129/C56BL/6) were kind gifts from Prof. Walter Wahli (University of Lausanne, Lausanne, Switzerland). Wild type mice (C57BL/6) were purchased from the InVivos Pte Ltd., Singapore. All animal procedures were reviewed and approved by the Nanyang Technological University Institutional Animal Care and Use Committee (IACUC, Project number: A0269), Singapore.

### 4.2. Cell Culture

Mouse melanoma cell line B16/F10 was cultured in Dulbecco’s modified Eagle’s medium (DMEM; Gibco, Carlsbad, CA, USA) supplemented with 10% fetal bovine serum (FBS; Gibco), 2 mM of l-glutamine (Gibco), 100 U/mL of penicillin and 100 µg/mL of streptomycin (Nacalai Tesque, Kyoto, Japan). Human Pulmonary Microvascular Endothelial Cells (HPMEC) cells were cultured in Endothelial Cell Medium-2 supplemented with endothelial cell growth medium bulletkits (Lonza, Cologne, Germany). All cell lines were maintained in 5% CO_2_ atmosphere at 37 °C.

### 4.3. Synthesis of 10h

Compound 10h (IUPAC: methyl 3-(*N*-(4-(isopentylamino)-2-methoxyphenyl)sulfamoyl)-thiophene-2-carboxylate; PPARβ/δ antagonist) was synthesised in-house (Shunsuke Chiba) according to as shown in Toth et al. 2012 [15]. The synthesis scheme can be found in Sng et al. 2018 [16].

### 4.4. RNA Extraction and Quantitative Real-time PCR

Total RNA was extracted using RNAzol RT (Molecular Research Centre, Cincinnati, OH, USA) and cDNA was synthesised using qScript cDNA Supermix (Quantabio, Beverly, MA, USA) according to manufacturer protocols. Quantitative real-time PCR was performed in a total of 20 µL volume containing SYBER green (PrimerDesign Precision, Camberley, UK) on a QuantiStudio 6 Flex Real-time PCR system. The primers used were as follows (Table 1):

### 4.5. Epithelial Mesenchymal Transition (EMT)

The B16/F10 cells (0.5 × 10^5^ cells/well) was seeded in a well of a 6-well plate and serum starved in serum free DMEM for 8 h before treatment with DMSO (0.05% *v*/*v*) as control or 10h (10 µM). After 72 h, the cells were harvested for western blot analysis to detect EMT marker proteins, MITF and MMP-9 expression or seeded for migration and invasion assays.

### 4.6. Measurement of Extracellular Melanin Content

For melanin content analysis, B16/F10 cells were cultured in phenol red-free DMEM (Gibco) containing 10% FBS. The cells were cultured with α-MSH at 0.1 µM (Sigma, St. Louis, MO, USA) treatment in the absence or presence of 10h for 3 days. The cultured cells or media were harvested, and pellets were dissolved in 1N NaOH (Merck, Kenilworth, NJ, USA) containing 10% DMSO (Sigma) at 80 °C for 1 h. The melanin content was measured at 475 nm wavelength using an ELISA plate reader (BioTek, Winooski, VT, USA).

### 4.7. Cell Adhesion and Tumour-Endothelial Assay

For cell adhesion assay, B16/F10 cells (6 × 10^4^ cells/well) were seeded in a fibronectin coated 96-well tissue culture plate and incubated for 30 min at 37 °C in a 5% CO_2_ incubator. Non-adherent cells were aspirated, and adherent cells were washed thrice with PBS. The remaining cells were then fixed with ethanol and stained with crystal violet. Excess dye was removed by washing with PBS and the dye absorbed by adherent cell nuclei was extracted with 0.5% Triton X-100. Quantification was by measuring the absorbance at 570 nm using an ELISA plate reader (BioTek).

For tumour-endothelial assay, B16/F10 cells were pre-treated with 0.05% DMSO or 10 µM 10h and pulsed with 25 µM Cell Tracker Green CMFDA (Invitrogen, Carlsbad, CA, USA) dye for 30 min and harvested to obtain a cell suspension. B16/F10 cells (2 × 10^4^) were seeded onto a monolayer of HPMEC and incubated for 15 min at 37 °C in a 5% CO_2_ incubator. Non-adherent cells were aspirated, and adherent cells were washed 3 times with PBS. Remaining adherent cells were fixed using 1% paraformaldehyde (PFA) for 15 min and the adherent cancer cells tagged with green signal were counted under an inverted fluorescence microscope (Nikon, Tokyo, Japan).

### 4.8. Trans-Well Migration and Invasion Assay

The cell migration assay was carried out using a transwell system with pore size of 8 µm (Corning Costar, Corning, NY, USA). The cells (0.8 × 10^5^ cells per well) were seeded into the upper chamber in serum free DMEM, while conditioned medium from NIH-3T3 was applied to the lower chamber. After 4 h incubation, migrated cells on the bottom surface of the membrane were fixed with 1% PFA, non-migrated cells on the top of the membrane were gently removed by cotton bud while the migrated cells at the bottom of the membrane were stained with DAPI. Migrated cells were counted in five random microscopic fields (original magnification, 10×). The cell invasion assay was carried out using a transwell system with pore size of 8 µm (Corning Costar, USA), coated with Matrigel (BD Biosciences, San Jose, CA, USA). Similarly, the cells (1 × 10^5^ cells per well) were seeded into the upper chamber in DMEM supplemented with 1% FBS, while medium with 10% FBS was applied to the lower chamber. After 24 h, invading cells were quantified as described above.

### 4.9. Trans-Endothelial Migration

1 × 10^5^ HPMEC cells were seeded in the upper chamber of a collagen-coated transwell insert and allowed to grow in complete endothelial medium to confluence (up to 4 days with P2 (HPMEC); fresh medium was added at day 2). Pre-treated B16/F10 cells with 0.05% DMSO or 10 µM 10h were pulsed with 25 µM CellTracker Green CMFDA dye (Invitrogen) for 30 min before being detached by 0.25% trypsin. 0.8 × 10^5^ of pre-treated B16/F10 cells were seeded onto the endothelial monolayer and allowed to migrate for 18 h at 37 °C in 5% CO_2_. Conditioned NIH-3T3 medium was used as a chemoattractant. The transwells were then fixed in 4% PFA and cells on the apical side of each insert were scraped off, and the transwell membrane mounted onto glass slides. Migration to the basolateral side of the membrane was visualized with a immunofluorescent microscope at 10× magnification. An average of five random fields were quantified using the NIH ImageJ analysis software (http://rsbweb.nih.gov/ij/). For trans-endothelial migration for HPMEC treated with 10h, the experimental design is similar to the above with the exception that the endothelial monolayer was treated with 0.05% DMSO or with 10h up to 4 days to confluence and green signal tagged B16/F10 cells were seeded onto the endothelial monolayer.

### 4.10. In Vivo Metastasis Assay

For experimental metastasis assays, pre-treated 5 × 10^5^ B16/F10 cells were injected into the tail vein of 6- to 8-week-old WT and *Pparβ/δ*^−/−^ mice as described [48]. After 2 weeks, the animals were sacrificed to harvest the lungs. The area or the number of metastases in the lungs was measured on photographs using the ImageJ software (http://rsbweb.nih.gov/ij/).

### 4.11. Extravasation Assay

1 × 10^6^ B16/F10 cells labelled with CellTracker Green CMFDA (Invitrogen) were injected into the tail vein of 6- to 8-week-old C57BL/6 female mice. Twenty-four hours later, mice were sacrificed, and lungs were harvested. Lung tissues were subjected to fixation using 4% PFA for 3 h and were cryoprotected in 15% sucrose and then 30% sucrose in PBS overnight. The lungs were then frozen using Shandon™ Cryomatrix™ embedding resin (Thermo Scientific, Waltham, MA, USA) and cryostat-cut in 6-μm thick sections. Immunofluorescent staining for blood vessels was conducted with anti-CD31 RFP conjugated primary antibody (1:100 dilution) (BD Biosciences, Bedford, MA, USA) and anti-DAPI (1:1000 dilution). The specimens were then examined using an upright widefield fluorescence microscope (Leica, Wetzlar, Germany).

### 4.12. Evans Blue Assay.

For evans blue extravasation assay, 200 µL of 0.5% Evans Blue Solution was injected into the tail vein of 6- to 8- week-old WT and *Pparβ/δ*^−*/*−^ mice. After 30 min, the mice were euthanized before perfused transcardially with 5 mL of PBS. The lungs were then excised, washed with PBS and allowed to dry at 37 °C for 48 h. The dry weights were recorded and 500 µL of formamide (Sigma, St. Louis, MO, USA) was added. The samples were then incubated for 24 h at 55 °C. The amount of extravasated evans blue was measured by the absorbance of 620 nm using an ELISA plate reader (BioTek).

### 4.13. Statistics

One-way ANOVA followed by Turkey’s post hoc analysis or two-tailed, unpaired Student’s *t*-test was used to determine whether the experimental samples were significantly different from the control samples. Differences were considered statistically significant at * *p* < 0.05; ** *p* < 0.01; *** *p* < 0.001.

## Figures and Tables

**Table 1:** Sequences of the forward and reverse primers utilized for gene expression analysis.

Target (Mouse)	Forward Sequence (5′–3′)	Reverse Sequence (5′–3′)
*Angptl4* (NM_020581.2)	GGGACCTTAACTGTGCCAAGAG	GGAAGTATTGTCCATTGAGATTGGA
*Cpt1a* (NM_013495.2)	ACACCATCCACGCCATACTG	TCCCAGAAGACGAATAGGTTTGAG
*Mmp9* (NM_013599.4)	GCCCTGGAACTCACACGACA	TTGGAAACTCACACGCCAGAAG
*Gapdh* (NM_001289726.1)	ACTGAGGACCAGGTTGTCTCC	CTGTAGCCGTATTCATTGTCATACC
